# Global trends in the clinical utilization of exosomes in dermatology: a bibliometric analysis

**DOI:** 10.3389/fmed.2024.1462085

**Published:** 2024-10-10

**Authors:** Shiqin Tang, Pai Cai, Huina He, Yanan Tian, Ruiying Hao, Xin Liu, Tingting Jing, Yanyan Xu, Xiaojing Li

**Affiliations:** ^1^School of Clinical Medicine, Hebei University of Engineering, Handan, Hebei, China; ^2^School of Information Engineering, Suihua University, Suihua, Heilongjiang, China; ^3^Affiliated Hospital of Hebei University of Engineering, Handan, Hebei, China; ^4^Handan Stomatological Hospital, Endodontics, Handan, Hebei, China; ^5^Hebei Key Laboratory of Immunological Dermatology, Handan, Hebei, China

**Keywords:** skin wounds, adipose-derived mesenchymal stem cells, exosome, bibliometrics, visual analysis

## Abstract

The arena of exosomal research presents substantial emerging prospects for clinical dermatology applications. This investigation conducts a thorough analysis of the contemporary global research landscape regarding exosomes and their implications for dermatological applications over the preceding decade. Employing bibliometric methodologies, this study meticulously dissects the knowledge framework and identifies dynamic trends within this specialized field. Contemporary scholarly literature spanning the last decade was sourced from the Web of Science Core Collection (WoSCC) database. Subsequent to retrieval, both quantitative and visual analyses of the pertinent publications were performed utilizing the analytical software tools VOSviewer and Citespace. A comprehensive retrieval yielded 545 scholarly articles dated from January 1, 2014, to December 31, 2023. Leading the research forefront are institutions such as Shanghai Jiao Tong University, The Fourth Military Medical University, and Sun Yat-sen University. The most prolific contributors on a national scale are China, the United States, and South Korea. Among the authors, Zhang Bin, Zhang Wei, and Zhang Yan emerge as the most published, with Zhang Bin also achieving the distinction of being the most cited. The International Journal of Molecular Sciences leads in article publications, whereas Stem Cell Research & Therapy holds the pinnacle in citation rankings. Theranostics boasts the highest impact factor among the periodicals. Current research hotspots in this area include Adipose mesenchymal stem cell-derived exosomes(ADSC-Exos), diabetic skin wounds, cutaneous angiogenesis, and the combination of biomaterials and exosomes. This manuscript constitutes the inaugural comprehensive bibliometric analysis that delineates the prevailing research trends and advancements in the clinical application of exosomes in dermatology. These analyses illuminate the contemporary research focal points and trajectories, providing invaluable insights that will inform further exploration within this domain.

## 1 Introduction

The skin, which constitutes the body’s most extensive physical, chemical, and immunological barrier, comprises the epidermis, dermis, and subcutaneous tissue (1). Chronic exposure to the external environment can precipitate skin instability and functional impairments, including pigmentary disorders, wounds, aging, and immunological dysfunctions resulting in skin diseases. Exosomes, small extracellular vesicles (EVs), possess diameters ranging from 40 to 150 nm. Their biogenesis entails three distinct stages: the formation of intraluminal vesicles via plasma membrane invagination, the inward budding of these vesicles to constitute multivesicular bodies (MVBs), and subsequently, the facilitation of effective intercellular communication through the release of these vesicle complexes, specifically exosomes (2–4). In recent years, stem cell therapy has witnessed substantial advancement across diverse research domains, attributable to its multifunctionality, enhancement of regenerative factor secretion, and inherent self-renewal properties. Exosomes are integral to the specialized functions of stem cells. As a cell-free therapeutic modality, exosomes facilitate the transfer of proteins, mRNA, and miRNA to regulate the functions of recipient cells, thereby circumventing the associated rejection risks of cell-based therapies and offering substantial potential in clinical dermatological applications.

According to a comprehensive review of the literature, the application of exosomes in dermatological settings has captivated the international scholarly community, yet it is noted that a systematic bibliometric analysis in this area is currently absent. Consequently, this study employs the Web of Science Core Collection (WoSCC) as its primary data source, a database esteemed as highly influential and ubiquitously acknowledged within academic circles. A comprehensive analysis of all pertinent literature in this domain is executed via the utilization of WoSCC. Furthermore, distinguished software tools, namely CiteSpace and VOS Viewer, are implemented for advanced bibliometric and visual analytics. This methodology seeks to elucidate the global research trajectory and emergent trends concerning the use of exosomes in dermatological applications, delivering a nuanced interpretation of advancements in this sphere to discern principal contributors and pivotal shifts, while concurrently shedding light on overarching research trends.

## 2 Research methodology and data sources

### 2.1 Research methodology

Unlike general reviews, bibliometrics is a quantitative research methodology that relies on publication, citation, and text data to describe and analyze the dynamics and progress of a discipline or field of study (5, 6). Bibliometric studies yield results that encompass descriptive statistics as well as information on keywords, texts, citations, authors, institutions, and references. These studies examine the frequency, relevance, centrality, and clustering of authors and textual data, thereby often being employed by researchers to explore evolutionary patterns, publication trends, author citation networks, and other aspects of a topic (7).

The bibliometric analysis software VOS Viewer offers various visualization views, utilizing keywords, co-institutions, co-authors, countries, and institutions. These views include Label Visualization, Density Visualization, Cluster Density Visualization, and Scatter Visualization. By examining the size of circles in the visualization map, one can identify high-frequency keywords, prolific authors, as well as prominent countries and institutions. The creation of these views does not necessitate additional computer programs and is characterized by ease of mapping, visually appealing views, and flexible data output (8). However, VOS Viewer has some limitations, such as a fixed style of visualization mapping and limited clustering information extraction. During cluster analysis, it can only segment ranges by color without forming cluster labels through key information extraction.

Another visualization software, Cite Space, focuses on assembling databases to analyze individual modules. Through similarity algorithms, it displays different temporal dimensions of the atlas, which are used to visualize temporal changes, development trends, and fundamental changes in the research field (9, 10). Additionally, data can be processed flexibly, such as through de-weighting and slicing, which can enhance the accuracy and efficiency of data analysis. However, there are some disadvantages, such as a relatively steep learning curve for beginners and software instability, which can hinder smooth data analysis.

In conclusion, while analyzing data using visualization software, it is essential to also emphasize the reading of traditional reviews. Only by effectively combining both approaches can we accurately screen data and gain insights into emerging hotspots in the field.

### 2.2 Data sources

**FIGURE 1:**
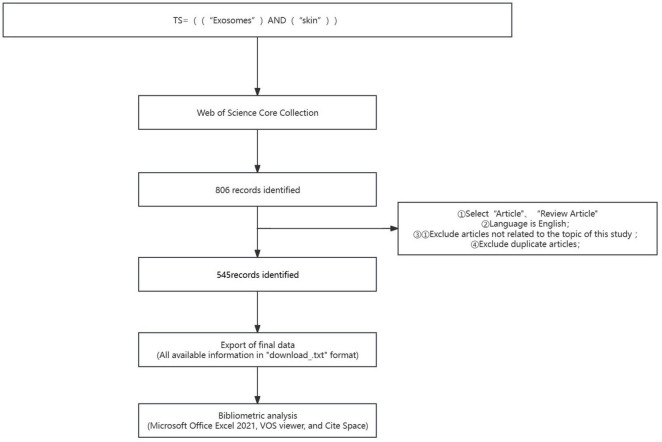
Illustrates the process of publication selection.

A structured query was initiated within the Web of Science Core Collection (WoSCC) database based on the stipulated criteria: ① The search theme was defined using the syntax: “TS = ((“Exosomes”) AND (“skin”))”. ②: The search was confined to content types “Article” and “Review Article”, with English specified as the language. ③: The temporal scope extended from January 1, 2014, to December 31, 2023. ④: The data retrieval culminated on March 11, 2024, to mitigate possible biases introduced by daily database updates. The corresponding link for this data retrieval is as follows:https://www.webofscience.com/wos/woscc/summary/3b781a47-f09f-4f09-95a2-816ef39da6c8-d38e046f/relevance/1. To refine the dataset, exclusion criteria were meticulously applied to discard literature not pertinent to the research topic. Initially, the search yielded 806 articles. Owing to potential duplicates or data inconsistencies derived directly from the search query, a preprocessing step was implemented to enforce the aforestated exclusion criteria prior to analysis. Following rigorous examination of each article, 545 valid publications were ascertained. The retrieved literature data was archived in TXT format, labeled as “full-text records and references”. The exported literature records encompassed titles, authors, affiliations of research institutions (research centers, universities, hospitals), abstracts, journals, publication dates, and references. Subsequently, this data was catalogued in an Excel database for detailed subsequent analysis (Figure 1).

## 3 Bibliometric analysis of the papers

### 3.1 Number of papers published

**FIGURE 2:**
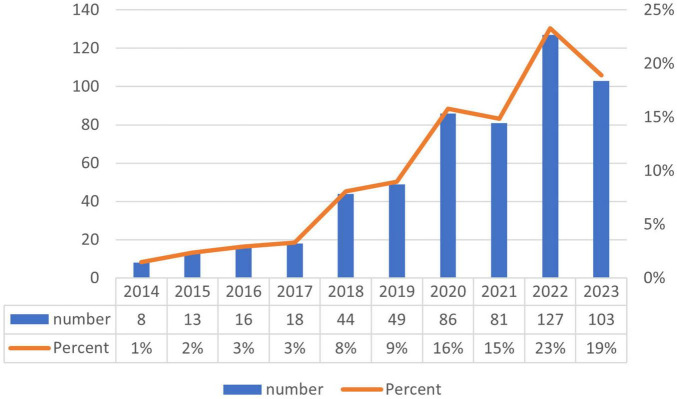
Annual publication volume in exosomes and skin-related research over the past decade.

Annual fluctuations in publication volumes can, to a significant degree, mirror changes in research themes, emerging trends, and prospective directions in development. Within the corpus of 545 articles analyzed in this study, contributions were made by authors spanning 54 countries and 958 institutions, collectively representing 21,752 researchers. These articles appeared in 220 distinct journals and cited 28,354 references across 3,824 different publications. Figure 2 delineates the temporal distribution of publications within the domain of exosome and skin-related research. Overall, the past decade has demonstrated a generally upward, albeit fluctuating, trend in this field, with 2014 recording the nadir of publication volume and reaching an apex in the year 2022.

### 3.2 Institutions and countries

This investigation engaged in a comprehensive analysis of publication volumes across 97 countries to ascertain which nations have substantially contributed to research in exosomes and skin-related fields over the preceding decade. Table 1 delineates the foremost 20 countries and institutions, predominantly situated across Europe (*N* = 10), Asia (*N* = 6), The Americas (*N* = 3), and Oceania (*N* = 1). Among these nations, China (*N* = 276, 45%) and the United States (*N* = 84, 14%) surfaced as the principal contributors in terms of publication volume, closely trailed by South Korea (*N* = 47, 8%).

**TABLE 1:** Top 20 countries and institutions in exosomes and skin-related research field.

Rank	Country	Counts	Percent	Institution	Counts	Percent
1	China	276	45%	Shanghai jiao tong univ	19	9%
2	USA	84	14%	Fourth mil med univ	17	8%
3	South Korea	47	8%	Sun yat sen univ	17	8%
4	Italy	33	5%	Huazhong univ sci & technol	15	7%
5	Germany	18	3%	Sichuan univ	15	7%
6	France	17	3%	Tongji univ	11	5%
7	Spain	16	3%	Jilin univ	10	5%
8	United Kingdom	16	3%	Wenzhou med univ	10	5%
9	Japan	15	2%	Jiangsu univ	9	4%
10	Iran	12	2%	Southern med univ	9	4%
11	Poland	11	2%	Cent soudi univ	8	4%
12	Australia	10	2%	Fudan univ	8	4%
13	Brazil	9	1%	Nanjing med univ	8	4%
14	Canada	8	1%	Zhejiang univ	8	4%
15	Singapore	8	1%	China med univ	7	3%
16	Switzerland	8	1%	Korea univ	7	3%
17	Austria	7	1%	Shandong univ	7	3%
18	India	7	1%	Xi an jiao tong univ	7	3%
19	Sweden	7	1%	Zunyi med univ	7	3%
20	Romania	6	1%	Chinese peoples liberal army gen hasp	6	3%

It is notable that among the top 20 global institutions catalogued in Table 1, Shanghai Jiao Tong University in China boasts the most substantial representation (N = 19). Closely following are esteemed institutions like The Fourth Military Medical University (N = 17, 8%) and Sun Yat-sen University (*N* = 17, 8%) from other countries.

**FIGURE 3:**
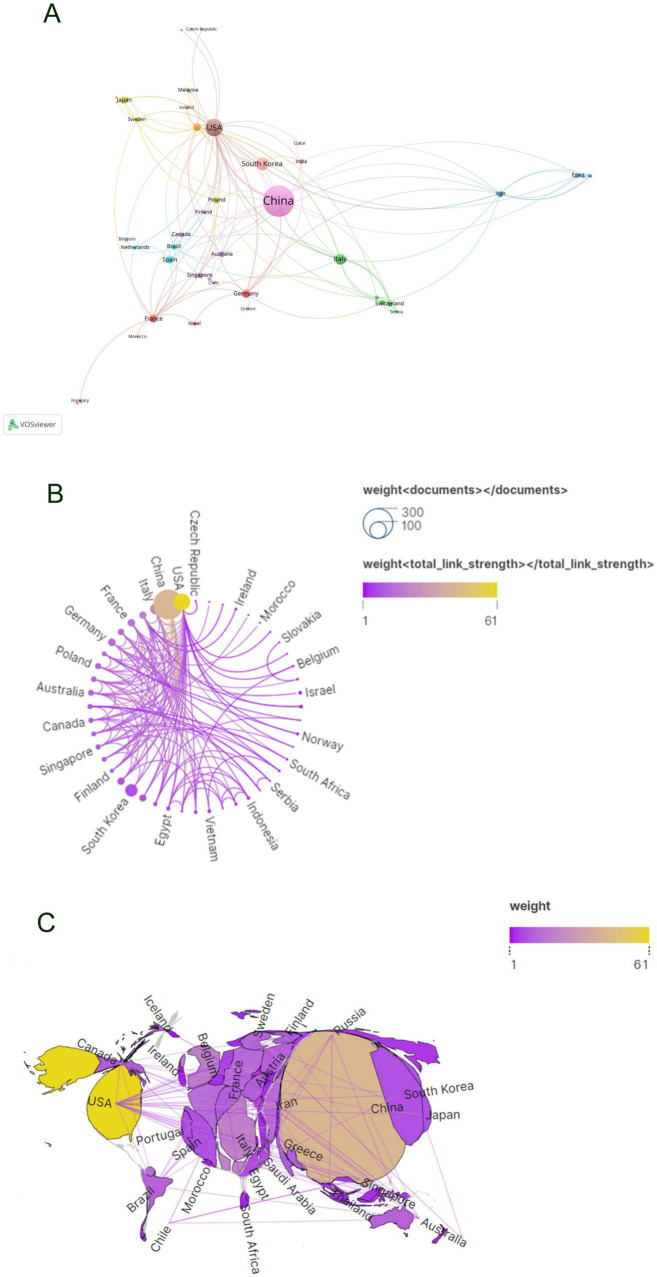
Visualization of countries in exosomes and skin-related research field **(A–C)**.

Subsequent to the initial analysis, visualizations were constructed for countries contributing three or more publications (Figures 3A–C), wherein links between nodes depict the robustness of collaborative ties, and thicker lines denote a higher frequency of co-authored publications between two nations. Node hues delineate distinct clusters. This graphical depiction illustrates China as the predominant cluster among the publishing nations in this area of research, unveiling a marked imbalance in publication distribution and a significant manifestation of the “head effect.”

**FIGURE 4:**
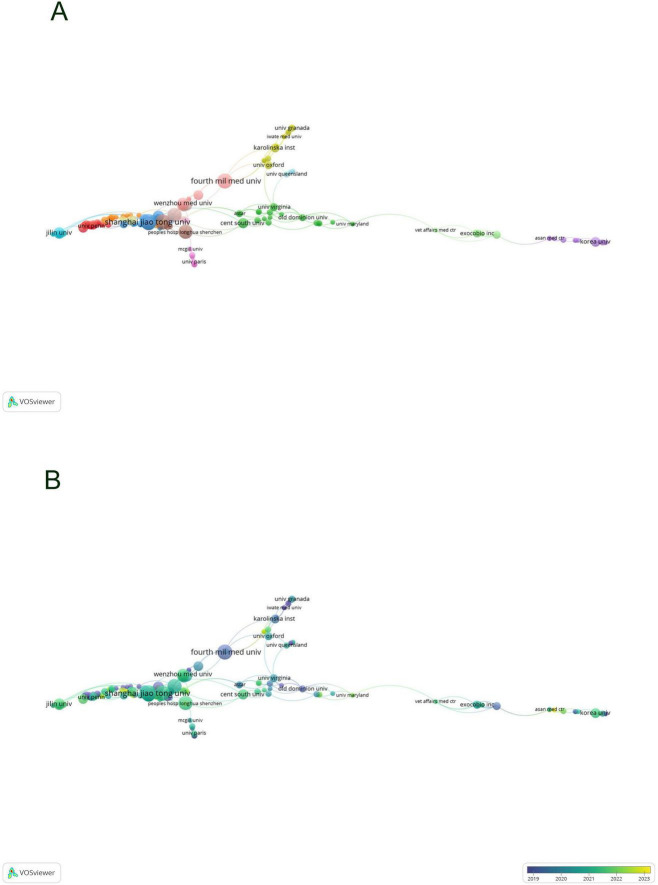
Visualization of institutions in exosomes and skin-related research field **(A,B)**.

By developing a visual network map of institutional collaborations (Figures 4A, B), our analysis uncovered a substantial partnership between Shanghai Jiao Tong University and Wenzhou Medical University, as well as Central South University—these institutions exhibiting the highest number of publications. Furthermore, collaborations were consistently observed among Zhejiang University, Sun Yat-sen University, Sichuan University, and Central South University. Institutions such as the University of Virginia demonstrated significant connections with Old Dominion University, University of Oxford, and The University of Queensland, each from distinct nations.

### 3.3 Author analysis

**FIGURE 5:**
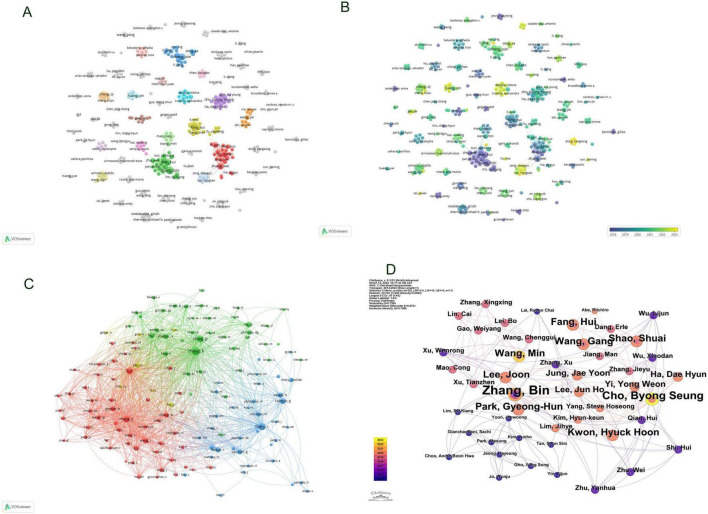
Visualization of Authors in exosome and skin-related research field **(A,B)**; Visualization of collaboratively cited authors in exosome and skin-related research field **(C,D)**.

The citation frequency of a scholarly article serves as a critical metric for gauging its academic impact. Among authors globally recognized for their prolific output, a total of 3,662 contributors engaged in research pertaining to exosomes and skin, with the top ten authors each having authored five or more papers. Within this cohort, five authors were cited more than 100 times, with the leading trio of cited authors comprising Zhang B (*N* = 171), Théry C (*N* = 127), and Zhang Y (*N* = 106) as documented in Table 2. Drawing upon this data, we devised a collaboration network diagram of authors who have each published a minimum of two papers (Figures 5A, B), encompassing 356 authors who met this criterion. Authors were distinctly clustered, with appearance frequencies denoted by color variations; each node in the diagram symbolizes an author, with node size mirroring the author’s publication volume. The lines interconnecting nodes signify the collaborative relationships among authors. Notably, Zhang Bin, Zhang Wei, Hu Dahai, Li Yan, and Shen Kuo exhibited the largest nodes, indicative of their extensive collaboration and prolific publication output. Further scrutiny revealed that upon applying a minimum citation threshold of 20, 137 authors satisfied the criteria, leading to the generation of a shared citation network diagram (Figures 5C, D). Among these, Zhang B emerged as the most eminent and extensively connected author, collaborating closely with authors including Zhang Bin, Cho Byong Seung, and Park Gyeong-Hun. These scholars actively partake in collaborations with their less prolific counterparts, thereby fostering the advancement of research related to exosomes and skin, and facilitating scholarly communication and exchange.

**TABLE 2:** Top 10 authors and collaboratively cited authors in exosome and skin-related research over the past decade.

Rank	Authors	Document	Co-cited authors	Citations
1	Zhang, Bin	9	Zhang, B	171
2	Zhang, Wei	7	Thery, C	127
3	Zhang, Yan	7	Zhang, Y	106
4	Qian, Hui	6	Li, X	105
5	Shi, Hui	6	Zhang, JY	105
6	Hu, Dahai	6	Raposo, G	86
7	Cho, Byong Seung	6	Lai, RC	76
8	Xu, Wenrong	5	Hu, L	70
9	Li, Yan	5	Kalluri, R	68
10	Fang, Hui	5	Wang, L	68

### 3.4 Journal analysis

The articles were disseminated across a total of 220 journals. The top ten journals are enumerated in Table 3. International Journal of Molecular Sciences (*N* = 43) spearheaded the selection with 855 citations and an average citation per article of 19.9, thereby securing the premier position. Following in close succession, Stem Cell Research and Therapy (*N* = 38) boasts the highest citation frequency, amassing a total of 1,933 citations. Frontiers in Immunology (*N* = 16) claimed the third position. The publication with the most elevated impact factor was Theranostics (IF = 12.4).

**TABLE 3:** Top 10 journals focusing on exosome and skin-related research in the past decade.

Rank	Journal	IF (2024)	Publications	JCR	Citations	Average citation/publication
1	International Journal of Molecular Sciences	6:2	43	Q2	855	19.9
2	Stem Cell Research & Therapy	7.5	38	Q2	1933	50.9
3	Frontiers in Immunology	7.3	16	Q2	361	22.6
4	Frontiers in Bioerigineering and Biotechnology	5.7	14	Q3	238	17.0
5	International Journal of Nanomedicine	8.0	14	Q2	429	30.6
6	Journal of Nanobiotechnology	10.2	14	Q1	329	23.5
7	Cells	6.0	12	Q2	515	42.9
8	Frontiers in Cell and Developmental Biology	5.5	12	Q2	237	19.8
9	Scientific Reports	4.6	12	Q2	664	55.3
10	Theranostics	12.4	9	Q1	1028	114.2

In accordance with data presented in Table 4, it is noteworthy that among the top ten most cited journals, six boast citations exceeding 500. Stem Cell Research & Therapy leads with the highest citation tally (*N* = 981), succeeded by International Journal of Molecular Sciences (*N* = 887), Scientific Reports (*N* = 702), PLOS One (*N* = 688), Journal of Investigative Dermatology (*N* = 603), Journal of Extracellular Vesicles (*N* = 539), Proceedings of the National Academy of Sciences (PNAS) (*N* = 430), Stem Cells (*N* = 403), Theranostics (*N* = 386), and Frontiers in Immunology (*N* = 382). The publication boasting the highest impact factor is the Journal of Extracellular Vesicles (IF = 16).

**TABLE 4:** Top 10 most cited journals in exosome and skin-related research in the past decade.

Rank	Co-cited journal	H index	IF (2024)	JCR	Co-citations
1	Stem Cell Research and Therapy	76	7.5	Q2	981
2	International Journal of Molecular Sciences	162	6.2	Q2	887
3	Scientific Reports	213	4.6	Q2	702
4	Plos One	332	3.7	Q3	688
5	Journal Of Investigative Dermatology	201	6.5	Q2	603
6	Journal Of Extracellular Vesicles	65	16	Q1	539
7	PNAS	771	11.1	Q1	430
8	Stem Cells	229	5.2	Q2	403
9	Theranostics	97	12.4	Q1	386
10	Frontiers in Immunology	124	7.3	Q2	382

**FIGURE 6:**
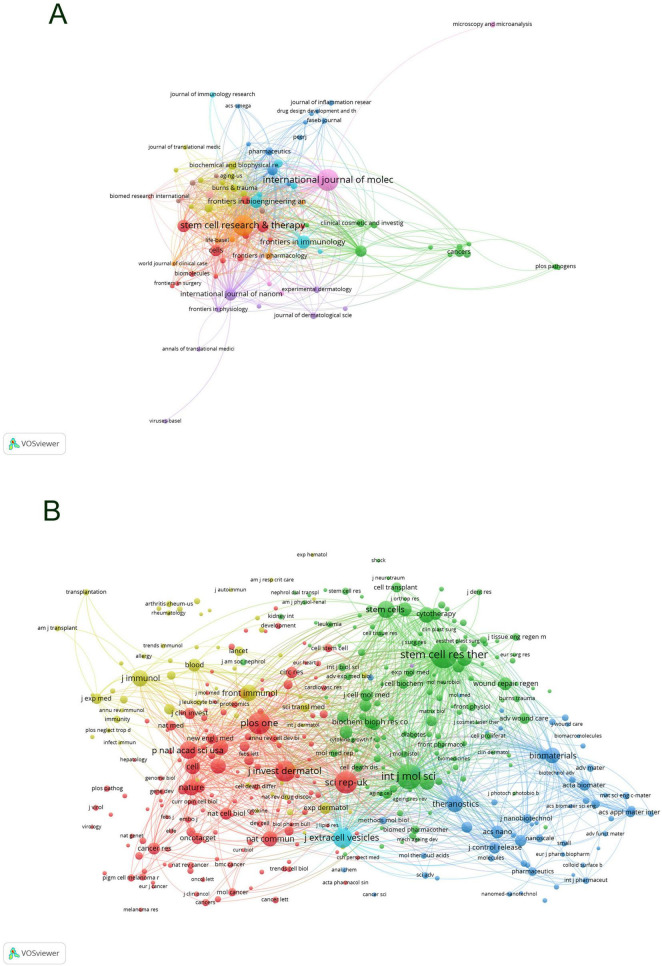
Collaboration network visualization of journals in exosome and skin-related research in the past decade **(A)** and co-citation collaboration network visualization of journals **(B)**.

Employing VOS Viewer, an initial total of 220 journals were filtered based on a minimum publication criterion of two, from which 82 journals were selected for further analysis. A network graph was generated of these journals (Figure 6A), showcasing International Journal of Molecular Sciences and Stem Cell Research & Therapy as nodes of high connectivity, indicative of their prolific publication output. Subsequently, further filtering was applied with a threshold of a minimum shared citation count of 20, leading to the inclusion of 3,824 journals in the shared citation network graph. Of these, 392 journals were incorporated as illustrated in Figure 6B. The graph segregated into four primary color clusters, with a prominent abundance of red and green nodes denoting significant interconnectivity among these clusters. Conversely, the pale yellow nodes were fewer in number compared to the blue nodes, suggesting an area for enhancement in the shared citation count within the pale yellow cluster, potentially due to the content and scholarly impact of the included publications.

**FIGURE 7:**
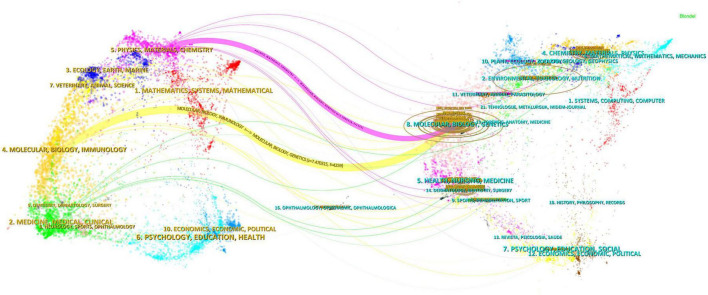
Dual mapping of journals in exosome applications in dermatology research over the past decade.

The dual-mapping overlay of journals delineates the relationships between the citing and the cited journals, with the left side representing the citing journals and the right side the cited journals. As illustrated in Figure 7, the yellow pathways denote significant citation relationships, illustrating that research disseminated in Molecular Biology and Genetics journals is predominantly cited by journals in the fields of Molecular Biology and Immunology. The purple citation trajectories suggest that outputs from Molecular Biology and Genetics journals are frequently referenced in disciplines such as Physics, Materials Science, and Chemistry.

### 3.5 Co-citation references and bibliographic bursts

From January 1, 2014, to December 31, 2023, a total of 28,354 co-citation references pertaining to research on exosomes and skin were recorded. Within the foremost 10 co-citation references (see Table 5), each reference was cited collectively no fewer than 50 times.

**TABLE 5:** Top 10 journals in exosome applications in dermatology research cited over the past decade.

Rank	Co-cited reference	Citations
1	Zhang B, 2015. stem cells, v33, p2158, doi: 10.1002/stem.l77 (13)	80
*2*	Zhang JY, 2015, j transl med, vl3, doi: 10.1186/s12967-015-0417- (12)	74
3	Valadi H, 2007, nat cell biol, v9, p654, doi: 10.1038/ncb159 (11)	67
4	Hu L, 2016, scirep-uk, v6. doi: 10.1038/srep3299 (53)	61
5	Thery C, 2018, j extracell vesicles, v7, doi: 10.1080/20013078.2018.153575 (54)	60
6	Kalluri R, 2020, science, v367, p640, doi: 10.1126/science.aau697 (55)	59
7	Raposo G, 2013, j cell biol, v200, p373, doi: 10.1083/jcb.20121113 (56)	59
8	Shabbir A, 2015, stem cells dev, v24, p1635, doi: 10.1089/scd.2014.031 (57)	58
9	Fang S, 2016, stem cell transl med, v5, p1425, doi: 10.5966/sctm.2015-036 (58)	56
10	Thery clotilde, 2006, curr protoc cell biol, vchapter 3. doi: 10.1002/0471143030.cb0322s3 (59)	52

**FIGURE 8:**
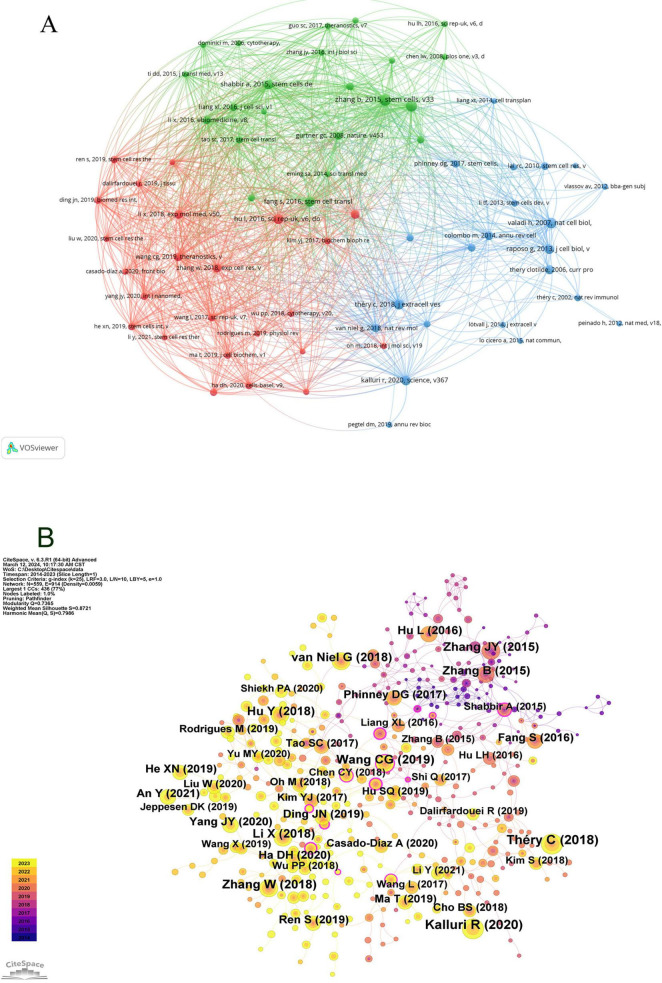
Visualization of co-citation references in exosome and skin-related research over the past decade.

Employing VOSviewer, a co-citation network view (Figure 8A) was constructed, selecting references that had garnered at least 20 citations. In Figure 8A, entities are represented in three distinct colors; red denotes the most prolific references, succeeded by green, and subsequently blue. Intriguingly, a heightened co-citation strength is observed between the red and blue categories. Larger circles within the graph signify higher citation counts, thereby reflecting the perceived scholarly impact of the references. Notable references including (11, 12, 13) demonstrate robust co-citation relationships.

Subsequently, utilizing CiteSpace configured with a temporal span from January 1, 2014, to December 31, 2023, and partitioning each year into individual time slices, with “references” designated as the node type, resulted in the generation of 559 nodes as per these stipulated parameters (Figure 8B).

**FIGURE 9:**
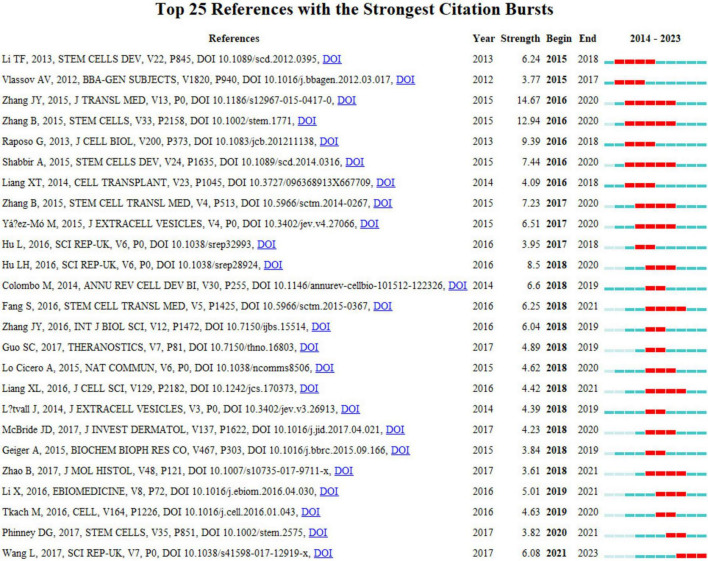
Top 25 co-cited references in exosome and skin-related research over the past decade.

The concept of a citation burst refers to the intense frequency with which a reference is cited by scholars within a specific field over a defined period. When a collection of articles is consistently cited, this phenomenon leads to the formation of concept clusters (14). In this analysis, CiteSpace identified 25 references exhibiting significant citation bursts. As depicted in Figure 9, references are organized according to their burst initiation year, with each respective bar symbolizing an individual year. The red lines denote a precipitous increase in citations for a particular reference during a specific year.

The most pronounced citation burst, registering a strength of 14.67, was recorded in the research conducted by Jieyuan Zhang’s team (12), focusing on “Exosomes released from human induced pluripotent stem cells-derived MSCs facilitate cutaneous wound healing by promoting collagen synthesis and angiogenesis.” Closely following this is the study by Bin Zhang’s team on “Huc MSC-Exosome Mediated-Wnt4 Signaling Is Required for Cutaneous Wound Healing,” which manifested a citation burst strength of 12.94 (13). It is particularly notable that both studies were authored by Chinese scholars, each demonstrating burst strengths exceeding 10.0, with sustained citation prominence from 2016 to 2020. The third-ranked article, by burst strength (9.39), is titled “Extracellular vesicles: exosomes, microvesicles, and friends.”

An incisive examination of the burst references presented in Figure 9 and Table 6 indicates that 18 of these articles pertain to investigations on the role of exosomes in skin healing, specifically those originating from mesenchymal stem cells (MSCs). Moreover, seven articles delve into elucidating the characteristics, mechanisms, and functional roles of exosomes. These analyses substantiate that the citation burst strengths of these 25 references span from 3.61 to 14.67, exhibiting a sustained impact lasting between two and five years.

**TABLE 6:** Main content of the top 25 co-cited journals in exosome and skin-related research over the past decade.

Rank	Strength	Main research content
1	6.2–5	Extracellular vesicles derived from human umbilical cord mesenchymal stem cells mitigate hepatic fibrosis.
2	3.77	Composition, biological functions, diagnostic and therapeutic potential of extracellular vesicles.
3	14.67	Extracellular vesicles derived from human mesenchymal stem cells promote collagen synthesis and angiogenesis to facilitate cutaneous wound healing.
4	12.94	Extracellular vesicles derived from mesenchymal stern cells convey Wnt4. thereby promoting wound healing.
5	9.39	A comprehensive review of the mechanisms, characteristics, and functions of extracellular vesicles.
6	7.44	Research on the promotion of wound healing by extracellular vesicles derived from mesenchymal stem cells.
7	4.09	The paracrine mechanisms of mesenchymal stem cell therapy.
8	7.23	Extracellular vesicles derived from human umbilical cord mesenchymal stem cells potentiate angiogenesis and facilitate vascular regeneration via the Wnt4/β-catenin pathway.
9	6.51	The biological characteristics and physiological functions of extracellular vesicles.
10	3.95	Extracellular vesicles derived from human adipose-derived mesenchymal stem cells expedite skin wound healing by optimizing the properties of fibroblasts.
11	8.5	The communication role of extracellular vesicles in diverse cell types.
12	6.6	The mechanisms, characteristics, and functions of extracellular vesicles.
13	6.25	Extracellular vesicle-derived microRNAs from umbilical cord mesenchymal stem cells inhibit the differentiation of myofibroblasts by suppressing the transforming growth faclor-beta/SMAD2 pathway involved in the wound healing process.
14	6.04	Extracellular vesicles derived from human endothelial progenitor cells facilitate skin wound healing through Erk1/2 signaling pathway activation.
15	4.89	Extracellular vesicles derived from platelet-rich plasma enhance re-epithelialization of chronic skin wounds in a diabetic rat model by activating YAP pathway.
16	4.62	Extracellular vesicles released by keratinocytes regulate melanocyte pigmentation deposition.
17	4.42	Exosomes secreted by mesenchymal stem cells facilitate endothelial cell angiogenesis through transfer of miR-125a.
18	4.39	Definition and function of extracellular vesicles.
19	4.23	Extracellular vesicles containing CD63, derived from bone marrow- mesenchymal stem cells, facilitate the extracellular transport of Wnt3a and enhance proliferation, migration, and angiogenesis in dermal fibroblasts.
20	3.84	Exosomes derived from human fibroblasts expedite wound healing in a mouse model of hereditary diabetes mellitus.
21	3.61	Extracellular vesicles derived from human amniotic epithelial cells accelerate wound healing and suppress scar formation.
22	5.01	Extracellular vesicles derived from human umbilical cord mesenchymal stem cells mediate the alleviation of bum-induced excessive inflammation through the modulation of miR-181c.
23	4.63	Exploration of the Characteristics, Functions, and Mechanisms of Extracellular Vesicles
24	3.82	Therapeutic Potential of Mesenchymal Stem Cell-Derived Extracellular Vesicles
25	6.08	Remodeling of Extracellular Matrix by Adipose-Derived Mesenchymal Stem Cell-Secreted Extracellular Vesicles: Promoting Scarless Skin Repair.

### 3.6 Keywords and research hotspots

**FIGURE 10:**
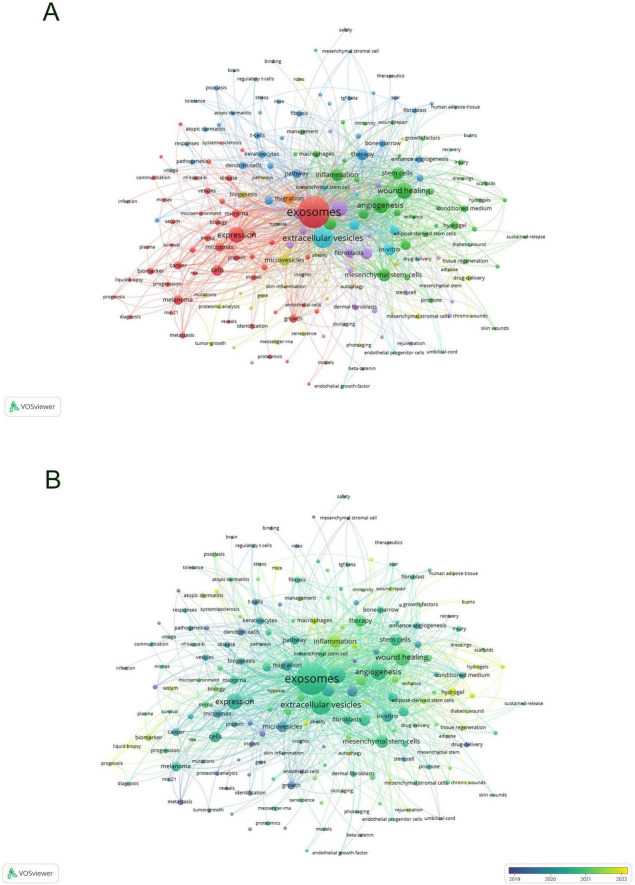
Visualization of keyword network in exosome and skin-related research **(A)** and overlay visualization of keywords **(B)**.

Keywords encapsulate the quintessence of scholarly texts, serving as pivotal access points for literature searches. An analysis of keywords within a specific field can reveal both current focal points and prospective trends. Of the 2,598 keywords subjected to analysis via VOS viewer, with a minimal occurrence threshold of five, 195 keywords surpassed this benchmark. Upon clustering and calculating the aggregate connection strength of these 195 keywords, a visualization manifesting keyword clusters was generated (Figure 10A), where larger circles denote higher keyword strength and denser lines between nodes indicate more frequent co-occurrence of keywords. In the overlaid visualization map (Figure 10B), color-coded temporal segments illustrate the evolution of keyword focal areas, with early-stage keywords depicted in navy blue and recent focal points in yellow-green. Detailed in Table 7, employing Citespace, the top 20 keywords were compiled, with the foremost three appearing over 100 times. Based on frequency analysis, we identify the global research emphases, underscored by interests in exosomes, extracellular vesicles, wound healing, and other relevant terms.

**TABLE 7:** Top 20 keywords with highest frequency in exosome and skin-related research in the past decade.

Rank	Frequency	Centrality	Year	Keywords
1	169	0.07	2014	Exosome
2	153	0.01	2016	Extracellular vesicles
3	104	0.08	2015	Wound healing
4	97	0.05	2014	Expression
5	93	0.03	2016	Mesenchymal stem cells
S	80	0.1	2014	Skin
7	74	0.06	2015	Angiogenesis
8	54	0.01	2018	Stem cells
9	50	0.03	2016	Proliferation
10	48	0.11	2014	*In vitro*
11	47	0.07	2015	Cells
*12*	44	0.15	2015	Differentiation
13	43	0.01	2017	Therapy
14	42	0.09	2015	Stromal cells
15	39	0.1	2017	Mechanisms
16	39	0.04	2017	Repair
17	38	0.02	2015	Microvesicles
18	36	0.05	2016	Migration
19	36	0.05	2018	Inflammation
20	30	0.05	2017	Regeneration

**FIGURE 11:**
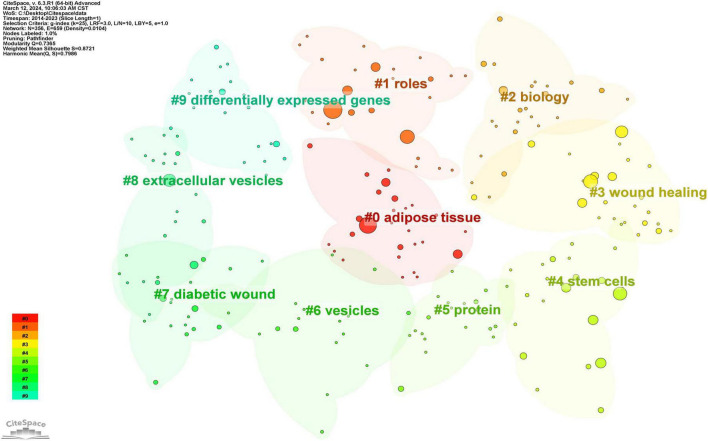
Clustering of keywords in exosome and skin-related research.

Subsequent to employing CiteSpace for the cluster analysis of keywords, depicted in Figure 11, keywords were systematically categorized into ten principal clusters, namely: #0 adipose tissue; #1 roles; #2 biology; #3 wound healing; #4 stem cells; #5 protein; #6 vesicles; #7 diabetic wound; #8 extracellular vesicles; and #9 differentially expressed genes. Each cluster, denoted by identifiers such as #0, #1, etc., represents a cohesive assembly, with larger clusters encompassing a more substantial number of constituents (15).

**FIGURE 12:**
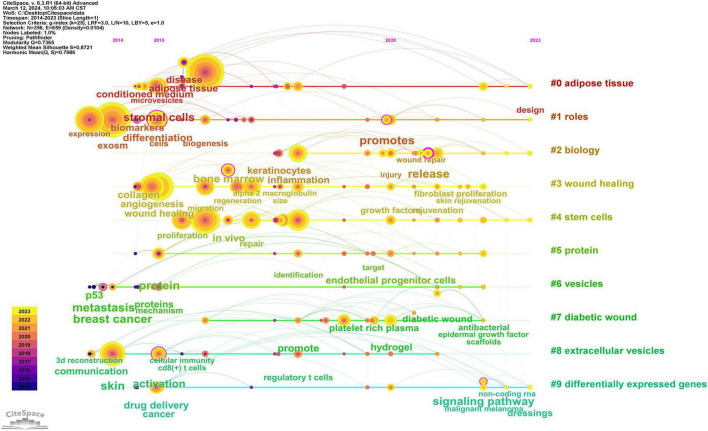
Timeline graph of key words in exosome and skin-related research.

By scrutinizing the timeline, one can discern the dynamic evolution of research focal points, as denoted by the keywords, spanning from January 1, 2014, to December 31, 2023. This timeline encapsulates the emergence and attenuation of pivotal themes, capturing the ascension and recession of specific topics. Keywords within identical clusters are aligned horizontally, arrayed in chronological order from left to right, illustrating the temporal progression from earlier to more recent developments. The prevalence of keywords within each cluster underscores the developmental magnitude and significance of that particular cluster in advancing research within the domain. Employing CiteSpace for a comprehensive keyword analysis, we constructed a temporal keyword timeline that delineates the evolving research hotspots and dynamic trends in the application of exosomes within dermatological studies. This visualization provides a chronological perspective on the field’s progression, thereby illuminating emerging focal areas and guiding future research directions. The clustering graph is evaluated using the Modularity Q and Weighted Mean Silhouette (S) indices to assess structural integrity and cohesion. This approach reveals a noteworthy division structure, indicated by a Q value greater than 0.3, and a reasonable clustering coherence, as suggested by an S value above 0.5 (16). The present analysis reveals that the keyword clustering module, quantified by a Modularity Q value of 0.7365, significantly exceeds the threshold of 0.3, and the Weighted Mean Silhouette (S) value of 0.8721, which also surpasses the critical value of 0.5, collectively suggesting a well-rationalized and distinctly defined clustering structure. In Figure 12, spanning the years 2020 to 2023, the predominant keywords encapsulate themes including disease, adipose tissue, conditioned medium, microvesicles, stromal cells, biomarkers, expression, exosome, cells, biogenesis, differentiation, collagen, angiogenesis, wound healing, migration, bone marrow, regeneration, alpha-2-macroglobulin, injury, wound repair, fibroblast proliferation, skin rejuvenation, growth factor, and rejuvenation. This comprehensive array highlights foundational and emergent areas of inquiry within the field.

## 4 Discussion

### 4.1 General information

In the present investigation, we extracted bibliographic data from the Web of Science Core Collection (WoSCC) and undertook a visualization analysis of this corpus utilizing tools such as CiteSpace and VOS Viewer. We explored the dimensions of the research findings, specifically examining annual publication volume, contributing authors, affiliated institutions, pertinent keywords, involved countries, publishing journals, and referenced sources. As depicted in Figure 2, an analysis of the overarching trend reveals a fluctuating yet ascendant trajectory in publication volume over the preceding decade, with a nadir in 2014 and an apex in 2022. Recent scholarly attention has coalesced around the domain of exosomes and dermatological studies, with China, the USA, and South Korea distinguishing themselves as frontrunners in research contributions within this arena. Notably, the country analysis reveals a pronounced top-heavy distribution, with a select few nations dominating the scholarly output, which suggests the presence of limited collaborative networks and a deficiency in proactive international cooperation. Enhanced international collaboration is imperative for the progression of this scientific field, as cooperative endeavors are pivotal in nurturing its growth and facilitating further development.

In the global institutional landscape, Shanghai Jiao Tong University emerges as the leader in publication volume, highlighting its substantial and enduring engagement with research in the realms of exosomes and dermatology. Journal ranking analysis illustrates that the International Journal of Molecular Sciences leads with the highest publication count (N = 43), amassing 855 citations and an average of 19.9 citations per article, thereby securing the premier position in the field. The most frequently cited journal is Stem Cell Research & Therapy(N = 981). Noteworthily, amongst both publishing and heavily cited journals, Theranostics boasts the highest impact factor (IF = 12.4).

Author Zhang, Bin emerges as the preeminent scholar in terms of both publication volume (N = 9) and citations (*N* = 171), affirming his status as a pivotal contributor within this research domain. Zhang engages in extensive collaboration with notable peers including Zhang, Wei, Wang, Xujie, Hu, Dahai, Han, Shichao, Su, Linlin, among others. This research team, led by Zhang, specializes in investigating the role of Mesenchymal Stem Cell-Derived Exosomes (MSC-Ex) in the arena of skin wound healing (17–20). Their investigations have elucidated that MSC-Ex significantly contributes to wound regeneration and cell proliferation via the activation of Wnt4, thus significantly enhancing the process of skin wound healing (13).

Zhang Wei, holding the global second rank in publication volume, has fervently pursued research focused on the healing of skin wounds and mitigation of scar formation through the study of exosomes derived from ADSC-Exos and human amniotic epithelial cells-derived mesenchymal stem cells (21–23). Among the top ten most cited references, the preponderance focuses on the role and mechanisms of mesenchymal stem cell-derived exosomes in skin wound repair, complemented by several studies delving into the general functions of exosomes. Consequently, the corroborated data substantiates that the international academic community is significantly engaged in investigating the role of mesenchymal stem cell-derived exosomes in skin wound repair and the intricate mechanisms involved.

### 4.2 Hotspots and frontiers

The analysis of high-frequency keywords facilitates the discernment of pivotal research hotspots and evolutionary trends within specific scientific domains (24). Utilizing keyword co-occurrence analysis has delineated the primary trajectories and research hotspots pertinent to exosomes and skin, revealing significant developments and thematic shifts. Employing keyword clustering and temporal analyses, not only were core keywords such as ‘exosome,’ ‘extracellular vesicles,’ and ‘microvesicles’ highlighted, but additional terms also surfaced, including ‘wound healing,’ ‘expression,’ ‘mesenchymal stem cells,’ ‘angiogenesis,’ and others, illustrating a rich tapestry of interconnected research areas. These keywords encapsulate the dynamic hotspots and emerging frontiers within the domain of exosome research and skin, underscoring the critical aspects under investigation.

Exosomes, defined as extracellular vesicles ranging from 30 to 150 nm in diameter, serve as critical conduits for intercellular communication within the skin’s cellular framework (25).

In recent years, mesenchymal stem cells (MSCs) have captured the focused attention of the global scientific community. Characterized by their self-renewal capabilities and multipotent differentiation potential, these cells have been extensively explored across diverse scientific disciplines. Mesenchymal stem cells primarily originate from embryonic stem cells (ESCs) and induced pluripotent stem cells (iPSCs) (26–29), demonstrating a remarkable ability to differentiate into various cell types including adipocytes, chondrocytes, and myocytes, contingent upon their tissue origin (30).

It is clear that mesenchymal stem cells, originating from a variety of sources, produce exosomes that exhibit distinct functions and characteristics. Exosomes derived from mesenchymal stem cells have been demonstrated to play pivotal roles in skin wound repair and tissue regeneration (31, 32),notably facilitating angiogenesis at injury sites (33), enhancing the speed of wound healing processes (34, 35), and are conveniently sourced from materials such as umbilical cord, adipose tissue, and bone marrow (25, 36).

Our analysis indicates that, over the past decade, exosomes sourced from ADSC-Exos have emerged as the predominant focus of research within the realm of skin-related applications. Particularly, the application of ADSC-Exos in diabetic wound repair has predominated, with a concentration on enhancing vascularization and protein synthesis. Numerous researchers have dedicated their efforts to augmenting wound healing rates by targeting specific differentially expressed genes through the deployment of exosomes derived from mesenchymal stem cells.

Skin wound healing represents a multifaceted reparative process, characterized by hemostasis, inflammation, proliferation, and tissue remodeling, and involves intricate interactions among diverse cytokines and components of the extracellular matrix (ECM). Vascular formation serves as a critical determinant of outcomes in diabetic wound healing (37). Exosomes sourced from ADSC-Exos have demonstrated potential in enhancing wound healing in diabetic patients by accelerating vascular formation, thereby amplifying the therapeutic potential for diabetic wounds (38, 39). Wang et al. (40) have engineered a bioactive hydrogel incorporating exosomes derived from ADSC-Exos, which facilitates enhanced vascular formation and expedited wound healing in diabetic patients. In their research, Wang et al. observed that the deployment of circ-Astn1-modified exosomes from ADSC-Exos significantly bolsters endothelial cell proliferation and wound healing in diabetic wound management (41). Research conducted by the Guo group demonstrated that miR-125b-5p, isolated from ADSC-Exos and identified via differential gene expression analysis, likely plays a pivotal role in targeting ACER2 for reparative functions in ischemic muscles associated with type 2 diabetes (42).

The use of exosomes in skin wounds mitigates the risks associated with cell transplantation and demonstrates significant potential in cell-free regenerative medicine. Recently, MSCs have been recognized for their crucial role in tissue regeneration and wound repair, facilitating angiogenesis, modulating ECM remodeling, skin barrier repair, and accelerating wound closure. Compared to other types of MSCs, ADSCs-Exos are more effective and can secrete many of the cytokines associated with wound repair while maintaining the characteristics of MSCs (43–46). Studies indicate that in diabetic skin wounds, ADSCs-Exos accelerate healing by promoting macrophage polarization towards the M2 type, enhancing anti-inflammatory cell counts, and stimulating fibroblast proliferation and migration, as well as tissue angiogenesis (47–49). Currently, numerous challenges persist in applying exosomes to cutaneous wound repair. The application of exosomes from various sources in wound treatment faces challenges due to their short duration, as they are rapidly cleared from the application site and have a short half-life in vivo, potentially limiting their long-lasting effects (50). This analysis suggests that future research will likely focus on extending the retention time of exosomes on wound surfaces by integrating them with biomaterials, without compromising their biological activity, to develop treatments for skin diseases using ADSCs-Exos (51, 52).

After conducting a comprehensive bibliometric analysis of the research field concerning EVs in relation to dermatological applications, notable contributions emerge from China, the United States, and South Korea. Herein lies a meticulous exploration of this phenomenon:

In recent years, China has significantly increased its investment in biomedical research, particularly in cutting-edge areas such as stem cell technology and EVs for wound healing. Government initiatives, including targeted financial aid, establishment of advanced research platforms, and attraction of international talent, have catalyzed rapid advancements in related studies. Moreover, China’s vast patient population and extensive clinical resources have provided an invaluable repository of samples and data. For instance, national initiatives such as the Major Science and Technology Projects aim to transcend key technological barriers and promote the translation and application of scientific achievements. Furthermore, the Science, Technology and Innovation 2030 Major Project includes regenerative medicine and precision healthcare, crucially supporting research on EVs and skin through policy backing and funding assurance. Furthermore, emphasis on research infrastructure, notably through the establishment of national key laboratories and engineering research centers, has facilitated deepened exploration into EVs and skin studies.

As a global leader in research and innovation, the United States has consistently maintained high levels of investment in biomedical research. With numerous world-class research institutions and universities, coupled with a robust funding framework, the United States provides significant backing for EVs and skin research. Efforts to accelerate clinical translation of research outcomes have enabled the swift application of relevant technologies and products. For example, initiatives such as the NIH funding programs encompass a broad spectrum, including EVs and skin research, significantly advancing United States innovations in this field. Additionally, stringent regulation and policy frameworks governing clinical trials underscore the United States’ government commitment to ensuring the effective translation of novel therapies, including EVs.

South Korea emerges as the third-highest contributor in this field, achieving notable milestones in biomedical research, particularly in dermatological aesthetics and anti-aging. The Korean government places a significant emphasis on biotechnology and regenerative medicine, supporting research institutions and enterprises through policy formulation and strategic planning. Moreover, active collaboration with international research institutions fosters global progress in EVs and skin research. For instance, initiatives like the K-BIO (Korea Biotechnology Industry Promotion Plan) bolster biotechnology and regenerative medicine sectors, facilitating expedited industrial growth. Comprehensive support, encompassing research grants, business assistance, and talent cultivation, underscores South Korea’s commitment to advancing EVs and skin research on a global scale. Participation in international research projects and hosting academic conferences further promotes transnational collaboration and enhances global contributions in EVs and skin research.

In summary, the significant contributions of China, the United States, and South Korea in extracellular vesicles and dermatological studies stem from their robust scientific capabilities, abundant research resources, and steadfast government support. Through continuous technological breakthroughs, particularly in the processes of EV extraction, purification, characterization, and functional studies, these countries have achieved pioneering results. Policy formulation and strategic planning have provided a substantial guarantee for the rapid development of this field. Looking ahead, as research deepens and technologies mature, the prospects for EV applications in dermatological care, wound healing, and anti-aging treatments are poised to expand further.

### 4.3 Limitation

In this investigation, the WoSCC was selected as the data source, recognized widely for its exemplary quality as a digital literature resource and its suitability for rigorous bibliometric analysis. However, it is pertinent to acknowledge certain limitations inherent in this study. Given that databases often harbor duplicate entries, subjective biases may influence the de-duplication process. The dataset was confined to English-language manuscripts categorized exclusively as “Articles” or “Review Articles” from the past decade, a constraint that may inadvertently exclude other seminal works. Despite these constraints, the analysis delineated herein continues to illuminate cutting-edge trends in exosome applications within dermatological research, offering substantial insights to experts in the arena.

## 5 Conclusion

Our study leveraged bibliometric analysis using VOSviewer and CiteSpace to evaluate geopolitical entities, academic institutions, authors, journals, and emerging research trends. Fluctuations in annual publication rates highlight varying levels of research emphasis among different nations. Notably, China, the United States, and South Korea exhibit high publication volumes, with China demonstrating a distinctive leadership role. However, other nations display potential for enhanced publication output. Furthermore, fostering closer international collaboration is crucial for elucidating novel therapeutic mechanisms.

Our analysis identifies current research hotspots, particularly extracellular vesicles derived from ADSC-Exos, with a focus on skin repair in diabetic wound healing. Prospective directions may involve integrating biomaterials with EVs to develop biocarriers that maintain EV bioactivity while enhancing durability, which is crucial for advancing EV applications in skin wound repair. Additionally, research trends in skincare and anti-aging are experiencing continued annual growth.

In conclusion, this study provides valuable insights for the global scholarly community engaged in advanced research on EV applications in dermatology.
